# Microbiota Variation Across Life Stages of European Field-Caught *Anopheles atroparvus* and During Laboratory Colonization: New Insights for Malaria Research

**DOI:** 10.3389/fmicb.2021.775078

**Published:** 2021-11-24

**Authors:** Lotty Birnberg, Eric Climent-Sanz, Francisco M. Codoñer, Núria Busquets

**Affiliations:** ^1^IRTA, Centre de Recerca en Sanitat Animal (CReSA, IRTA-UAB), Barcelona, Spain; ^2^ADM-Biopolis, Parc Cientific Universitat de València, Paterna, Spain

**Keywords:** *Anopheles atroparvus*, field-caught, laboratory colonization, 16S rRNA, microbiota, European mosquitoes

## Abstract

The potential use of bacteria for developing novel vector control approaches has awakened new interests in the study of the microbiota associated with vector species. To set a baseline for future malaria research, a high-throughput sequencing of the bacterial 16S ribosomal gene V3-V4 region was used to profile the microbiota associated with late-instar larvae, newly emerged females, and wild-caught females of a sylvan *Anopheles atroparvus* population from a former malaria transmission area of Spain. Field-acquired microbiota was then assessed in non-blood-fed laboratory-reared females from the second, sixth, and 10th generations. Diversity analyses revealed that bacterial communities varied and clustered differently according to origin with sylvan larvae and newly emerged females distributing closer to laboratory-reared females than to their field counterparts. Inter-sample variation was mostly observed throughout the different developmental stages in the sylvan population. Larvae harbored the most diverse bacterial communities; wild-caught females, the poorest. In the transition from the sylvan environment to the first time point of laboratory breeding, a significant increase in diversity was observed, although this did decline under laboratory conditions. Despite diversity differences between wild-caught and laboratory-reared females, a substantial fraction of the bacterial communities was transferred through transstadial transmission and these persisted over 10 laboratory generations. Differentially abundant bacteria were mostly identified between breeding water and late-instar larvae, and in the transition from wild-caught to laboratory-reared females from the second generation. Our findings confirmed the key role of the breeding environment in shaping the microbiota of *An. atroparvus*. Gram-negative bacteria governed the microbiota of *An. atroparvus* with the prevalence of proteobacteria. *Pantoea*, *Thorsellia*, *Serratia*, *Asaia*, and *Pseudomonas* dominating the microbiota associated with wild-caught females, with the latter two governing the communities of laboratory-reared females. A core microbiota was identified with *Pseudomonas* and *Serratia* being the most abundant core genera shared by all sylvan and laboratory specimens. Overall, understanding the microbiota composition of *An. atroparvus* and how this varies throughout the mosquito life cycle and laboratory colonization paves the way when selecting potential bacterial candidates for use in microbiota-based intervention strategies against mosquito vectors, thereby improving our knowledge of laboratory-reared *An. atroparvus* mosquitoes for research purposes.

## Introduction

Microorganisms that permanently or transiently reside in mosquitoes are collectively known as microbiota (Villegas and Paolucci, 2014). Bacteria (commensal and/or endosymbiotic), protists, viruses, and fungi, which are the main representatives of this consortium, can be horizontally acquired (i.e., venereal transmission, sharing of environmental/food sources) and/or maternally transferred (Bian et al., 2013; Eleftherianos et al., 2013; Gendrin and Christophides, 2013; Bili et al., 2016). Despite being found colonizing the mosquitoes’ midgut epithelia, hemolymph, salivary glands, and gonads (Dillon and Dillon, 2004; Minard et al., 2013; Villegas and Paolucci, 2014), the midgut microbiota has been the most extensively studied. The midgut microbiota is primarily shaped by the environment (Gendrin and Christophides, 2013; Dennison et al., 2014; Hegde et al., 2018) and varies dynamically throughout the mosquito’s life cycle (Duguma et al., 2015). During larval development, immature stages ingest organic matter, detritus, and microorganisms from their aquatic habitat (Merrit et al., 1992) and acquire a considerable fraction of their microbiota (Wang et al., 2011). Only those microbes that withstand and adapt to the midgut’s microhabitat could be passed through transstadial transmission, from larvae to adults, and may persist in mosquito populations as part of the indigenous microbiota (Pumpuni et al., 1996). In adult mosquitoes, diverse dietary regimes (e.g., plant sap and nectar or blood) may alter the composition of the microbiota and incorporate diversity into the microbial communities (Rani et al., 2009). In fact, descriptive studies that have characterized the microbiota of several field populations and/or laboratory colonies of culicid mosquitoes have suggested geographical, species, sex, and even individual variation (Yadav et al., 2015; Akorli et al., 2016; Muturi et al., 2016; Bascuñan et al., 2018; Rodriguez-Ruano et al., 2020; Saab et al., 2020; Tainchum et al., 2020; Silva et al., 2021).

The microbiota plays an essential role in relevant physiological traits of diverse vector mosquitoes, such as larval development (Chouaia et al., 2012; Coon et al., 2014; Martinson and Strand, 2021), mosquito lifespan (McMeniman et al., 2009; Wei et al., 2017; Mancini et al., 2020), fecundity, and blood digestion (Gaio et al., 2011; Gnambani et al., 2020). Moreover, microbiota has been involved in both infection susceptibility of the mosquito and vector competence. However, its role and mechanisms are diverse and extensive with some commensal bacteria and/or endosymbionts able to produce “anti-pathogen molecules” or activate cross-reactive innate immune responses (Dong et al., 2009; Moreira et al., 2009; Cirimotich et al., 2011; Ramirez et al., 2012; Bai et al., 2019).

In recent years, vector-borne disease research has focused its efforts on studying multiple aspects of mosquito–microbiota–pathogen interactions for the development of novel and more effective control strategies. Conventionally, in such studies, laboratory breeding of mosquito vectors has been a useful tool for obtaining large numbers of experimental individuals and controlling experimental conditions (Romoli and Gendrin, 2018). However, outcomes may not necessarily represent what might occur in the wild (Akorli et al., 2019) due to changes in the fitness of the mosquito and its immune system as consequence of the reduction in microbial diversity as previously reported in laboratory-colonized specimens (Rani et al., 2009; Duguma et al., 2015; Dada et al., 2020). Therefore, a better understanding of field-acquired microbiota during the laboratory colonization of sylvan mosquito populations is essential to set baselines for functional studies.

On the European continent, sibling species of the *Anopheles maculipennis* subgroup are considered the primary vectors of *Plasmodium* parasites, which are the causative agents of malaria (Sinka et al., 2010). Among them, *Anopheles atroparvus* van Thiel, 1927 is still one of the most widely distributed species, capable of transmitting local strains of both *Plasmodium vivax* and *Plasmodium falciparum* (Jetten and Takken, 1994; Bueno-Mari and Jiménez-Peydró, 2012), as well as imported strains of *P. vivax* (Daskova and Rasnicyn, 1982) and *Plasmodium ovale* (Garnham et al., 1954). Following the eradication of malaria from Europe, the study of its vectors suffered substantial decrease, accompanied in turn by a subsequent information gap on the biology of local populations of *Anopheles* mosquitoes. Currently, sporadic outbreaks of autochthonous malaria transmission (Santa-Olalla et al., 2010; Danis et al., 2011; Arends et al., 2013), in addition to an increase in the number of imported cases (Piperaki and Daikos, 2016) and the prognostics of resurgence in the continent due to globalization and climate change (Hertig, 2019), have awakened new interests in their study.

To date, the microbiota of *An. atroparvus* has not been analyzed, and for this reason, taking into consideration malarial research in Europe, the present work aimed to (i) identify the bacterial communities associated with a sylvan Mediterranean population of *An. atroparvus* and (ii) assess the influence of laboratory breeding on the structure (diversity and composition) of the mosquito’s natural microbiota. To accomplish these goals, the microbiota profile of a local population of *An. atroparvus* from the Ebro Delta, a former malaria transmission area of Spain, was characterized using high-throughput sequencing of the bacterial 16S ribosomal gene V3-V4 region. Bacterial community composition was identified in late-instar larvae, newly emerged females, and field-caught females. The contribution of water from the natural breeding site to the microbial diversity of this anopheles population was evaluated. Finally, the composition of the microbial communities was assessed throughout the laboratory colonization process, from both the sylvan population and those over the second, sixth, and 10th generations produced under controlled laboratory conditions.

## Materials and Methods

### Experimental Design

To characterize the microbiota of an *An. atroparvus* population in its original habitat and to then evaluate its evolution over 10 generations under controlled laboratory conditions, four time points were set: sylvan (T0), and second (F2), sixth (F6), and 10th (F10) generation produced in the laboratory. From the sylvan environment, third- and fourth-instar larvae (L), newly emerged females (E), and wild-caught females (F0) were sampled. From laboratory, F2, F6, and F10 7− to 9-day-old females that had been sugar-fed (sterile 10% sucrose solution *ad libitum*), but had never been blood-fed, were selected. To identify the contribution of breeding water to the bacterial community composition in this mosquito population, water from the natural breeding site (W) was sampled.

### Sample Collection and Processing for Microbiota Characterization

From July to September 2017, rice paddies in the municipality of Amposta (Ebro Delta, Spain) (40°42′32.5686″N, 0°35′12.2814″E) were visited once a week for the collection of immature stages and adult indoor catches. Late-instar larvae (L3–L4) and pupae were collected using the conventional dipping technique and were transported live to the laboratory in sterile plastic containers with water and substrate from their original breeding site. Additional water samples were collected at the same depth where larvae were found and transported to the laboratory in sterile plastic containers at 4°C for preservation. Female and male anophelines were captured inside an unused shed using mouth aspirators (John W. Hock Company, Gainesville, FL, United States) and placed inside sterile 30 × 30 × 30 cm BugDorm (BioQuip, Rancho Dominguez, CA, United States) insect rearing cages for transportation. At the entomology laboratory from the *Institut de Recerca i Tecnologia Agroalimentaries – Centre de Recerca en Sanitat Animal* (IRTA-CReSA) in Barcelona, wild-caught mosquitoes were colonized in the laboratory and bred as previously described (Birnberg et al., 2020).

To generate samples for microbiota analysis (Table 1), (i) larvae were pooled, (ii) pupae were transferred to mosquito breeders (BioQuip, Rancho Dominguez, CA, United States) containing water and substrate from their breeding site for adult emergence—only newly emerged females from the first 48 h were used, and (iii) wild-caught, second, sixth, and 10th generation females were frozen and pooled according to origin. Water samples were preserved at −80°C until DNA isolation.

**TABLE 1:** Sample selection per time point for microbiota analysis of *Anopheles atroparvus* from the Ebro Delta and along laboratory colonization.

**Time point**	**Sample type**	**Abbreviation**	**No. of pools**	**No. specimens per pool or volume (mL)**
T0	Water from breeding sites	W	3	100
	Larvae (L3–L4)	L	3	20
	Newly emerged females	E	3	20
	Adult field females	F0	3	20
F2	Adult females (second lab generation)	F2	3	20
F6	Adult females (sixth lab generation)	F6	3	20
F10	Adult females (10th lab generation)	F10	3	20

*F2, F6, and F10 correspond to 7- to 9-day-old females that had been sugar-fed (sterile 10% sucrose solution *ad libitum*), but had never been blood-fed.*

To eliminate any possibility of contamination during specimen handling, samples were surface sterilized as follows: first, one rinse in sterile water for 1 min, two consecutive washes in 70% ethanol for 5 min, one 5-min wash in a 10-fold dilution of commercial bleach [active chlorine (37 g/l initial concentration]), and a final rinse in sterile water for 1 min. Adult females were sterilized individually, while larvae were sterilized in pools. All samples were preserved at −80°C until molecular processing.

### DNA Extraction From Water Samples and Larvae and Mosquito Pools

Firstly, larvae and mosquito pools were homogenized using zirconia beads in a FastPrep-24^*TM*^ 5G (MP Biomedicals GmbH, Eschwege, Germany) bead beating grinder and lysis system. Then, genomic DNA from these samples was isolated with the QIAamp DNA Mini Kit (Qiagen, Hilden, Germany) following the protocol for Gram-positive bacteria in which lysozyme (Sigma) was added for enzymatic lysis. DNA from breeding water was extracted using the DNeasy PowerWater Sterivex Kit (Qiagen, Hilden, Germany) according to the manufacturer’s instructions. DNA was purified using the DNeasy Blood and Tissue kit (Qiagen, Hilden, Germany) and its quality and concentration evaluated using the NanoDrop spectrophotometer (Thermo Scientific, Waltham, MA, United States).

### 16S Ribosomal RNA Gene Sequencing and Bioinformatics Analysis

To generate sequencing libraries, the hypervariable region V3-V4 of the bacterial 16S ribosomal RNA (rRNA) gene was amplified (Klindworth et al., 2013) and the Illumina 16S metagenomics sequencing library preparation protocol (Illumina, Inc., San Diego, CA, United States) was followed. The quality of all libraries was verified individually using the Quant-iT PicoGreen dsDNA Assay kit (Invitrogen, Carlsbad, CA, United States), normalized, and equimolarly pooled in a single library pool. On an Illumina MiSeq platform, samples were paired-end sequenced using the MiSeq Reagent Kit v3 (2 × 300 cycles). Nuclease-free water and the ZymoBIOMICS^*TM*^ Microbial Community Standard (Zymo Research Corp., Irvine, CA, United States) were included as contamination and amplification controls, respectively, and treated as regular samples. Raw sequencing datasets retrieved by this study were deposited in the National Center for Biotechnology Information (NCBI) Sequence Read Archive (SRA) under the Bioproject #PRJNA660574.

After sequencing, raw reads (R1/R2) were merged in PEAR V.0.9.1 software^1^ applying the default parameters and specifying a 70-nt sequence overlap on each end. Then, adapters were identified and removed from the merged sequences using Cutadapt v1.8.1 (Martin, 2011) and sequences shorter than 100 bp were eliminated from the dataset to reduce erroneous taxonomic associations. Finally, low-quality sequences (*phred* score lower than Q20) were eliminated using the BBMap v38 Reformat package. After quality filtering, chimera sequences were identified and eliminated in “Uchime” (Edgar et al., 2011) by comparing the clean sequences to the ChimeraSlayer database. To assemble operational taxonomic units (OTUs), good-quality sequences with at least 97% similarity were clustered in the Cluster Database at High Identity with Tolerance (cd-hit) software v2.6.8 (Li et al., 2002). For OTU annotation, assemblies were compared to the 16S rRNA gene sequence reference (RefSeq) database of the NCBI and the closest hit was reported. Taxonomy summaries with relative abundances at phylum, family, genera, and species levels were generated.

### Data Analyses

Diversity analyses, ordination methods, and differential analyses for microbiota composition were performed in “R” v3.6.0 (R Core Team, 2016) statistical software. Prior data analyses, spurious OTUs (one sequence present in only one sample) were eliminated, and count matrices were rarefied at 20,465 sequences per sample using the “phyloseq” package as previously described (Weiss et al., 2017). In the “vegan” package for community ecological analyses (Okasen et al., 2020), alpha diversity was estimated by calculating the microbial/OTU richness and Shannon and Simpson indices. To assess the variation between sample types (i.e., breeding water (W), larvae (L), newly emerged (E) and wild-caught (F0) females, and second (F2), sixth (F6), and 10th (F10) generation laboratory females), an analysis of variance (ANOVA) was conducted. Differences between bacterial communities among sample types were evaluated by a principal coordinate analysis (PCoA) based on a Bray–Curtis dissimilarity matrix, and the significance of these associations was tested with a PERmutational Multivariate ANalysis Of VAariance (PERMANOVA) using 1,000 permutations. Data distribution was visualized in “ggplot” (Wickham, 2009). To measure and compare the uniqueness of bacterial communities from each sample type and assess their input to the diversity between groups, a local contribution to beta diversity (LCBD) test was executed (Legendre and De Cáceres, 2013). For differential abundance analysis, in the “DESeq2” package (Love et al., 2014), a generalized linear model (GLM) for fixed effects was generated using the negative binomial family between pairs of samples (W/L, L/E, E/F0, F0/F2, F2/F6, and F6/F10). Then, a Wald test was performed and the Benjamini and Hochberg false-discovery rate (FDR) correction was used for *p-value* adjustment (Benjamini and Hochberg, 1995). Bacterial taxa present in at least 50% of the samples of each group (i.e., W, L, E, or F*x*) with an average number of normalized sequences (BaseMean) higher than 10 that presented an adjusted *p-value* lower than 0.05 were considered as differential taxa. The logarithm 2 of the relative change (log_2_FC) of each bacterial group at the genus level was calculated to estimate the abundance of differential bacteria per pair of samples. To determine the contribution of natural breeding water to the microbiota of *An. atroparvus* and identify the bacteria that may persist as a result of transstadial transmission and/or over 10 generations under controlled laboratory conditions, the unrarefied OTUs were used to identify shared and unique genera. Common genera between two sample types, which were present in at least one pool (out of three) from each group, were considered as “shared.” Sample interactions were then represented with Venn diagrams. Finally, to describe the core microbiota, meaning the bacteria stably associated with a certain mosquito species in different mosquito stages (i.e., L, E, F0–F10), those genera identified in two pools out of three with at least 10 reads per each sample type were selected.

## Results

### Sequencing Data Output Summary and Taxonomic Assignations

High-throughput sequencing of the bacterial 16S rRNA gene V3-V4 region of *An. atroparvus* (sylvan and laboratory) and its natural breeding water generated a total of 1,364,231 raw reads. After quality filtering and chimeric sequence removal, reads per sample ranged from 20,465 to 107,148. In total, 1,082,199 clean sequences were used to assemble 20,462 different OTUs, of which 80% were successfully annotated and distributed into 24 phyla, more than 300 families, and nearly 1200 genera (Supplementary File 1). At phylum level, *Proteobacteria*, *Bacteroidetes*, *Actinobacteria*, *Firmicutes*, *Verrucomicrobia*, *Planctomycetes*, and *Cyanobacteria* accounted for 94% of the total microbiota. *Proteobacteria* was identified as the most abundant phylum gathering, by itself, 52% of the overall OTUs (Supplementary Figure 1A). At lower taxonomic levels, OTUs were distributed in several low abundant taxa with *Pseudomonadaceae* (7%), *Flavobacteriaceae* (4%), *Comamonadaceae* (4%), and *Acetobacteraceae* (4%) being the most abundant families and *Pseudomonas* (6%), *Asaia* (4%), and *Flavobacterium* (3%) being the most representative genera (Supplementary Figures 1B, C). Rarefaction curves in almost all samples, except for water, reached the plateau, implying that most of the bacterial diversity was captured (Supplementary Figure 2).

Negative and positive controls yielded 15 and 10,000 sequences, respectively. Since the few OTUs from the negative control exhibited low identities and none of these were detected in any of the studied samples, laboratory contamination was discarded. Likewise, since only the expected bacteria were identified in the microbial standard, taxonomic outcomes were verified.

### *An. atroparvus* Immature Stages Harbor More Diverse Bacterial Communities Than Adult Females

**FIGURE 1:**
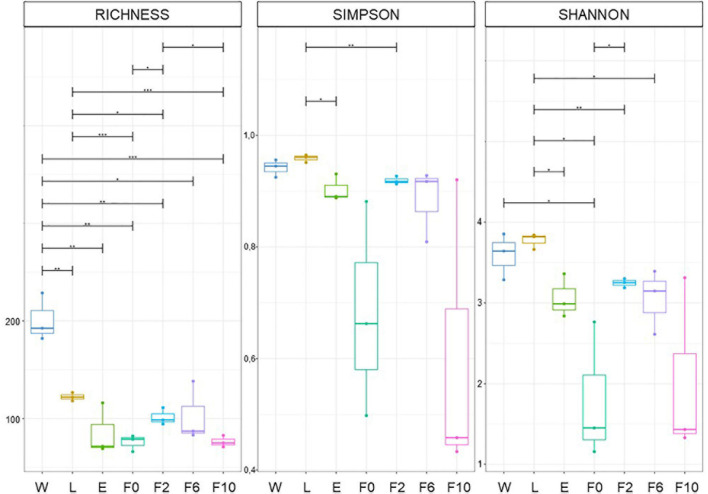
Differences in bacterial community structure. OTU richness and Simpson and Shannon indices estimated at family level. Sample types: W, breeding water; L, larvae; E, newly emerged females; F0, wild-caught females; F2, F6, and F10, laboratory-reared females from the second, sixth, and 10th generations, respectively. Boxes represent the interquartile range within each group. The line that divides the box corresponds to the median and dots, to minimum and maximum scores. Analysis of variance (ANOVA) significance levels: *p = 0.01–0.05; **p = 0.001–0.01; ***p < 0.001.

Diversity indices revealed that the structure (diversity and composition) of bacterial communities in breeding water, as well as those in sylvan and laboratory-reared *An. atroparvus*, varied according to group of origin (i.e., breeding water (W) larvae (L), newly emerged (E) and wild-caught (F0) females, and second (F2), sixth (F6), and 10th (F10) generation laboratory females). Pairwise ANOVA comparisons of OTU richness and Simpson (1-*D*) and Shannon (*H*) indices provided first-hand evidence of this variation. At all taxonomic levels analyzed (i.e., family, genera, and species) (Figure 1 and Supplementary Figures 3, 4), OTU richness was significantly higher in breeding water (W) than in larvae (L) and adult mosquito samples (E and F0–F10) (*p* < 0.05). Within sylvan and laboratory environments, significant differences were found between L and F0 (*p* = 0.001–0.01) and between F2 and F10 (*p* = 0.001–0.05), respectively. Simpson and Shannon indices showed that, among the studied biological samples, L was the most diverse and evenly distributed, while F0 and F10 were the least diverse and highly uneven samples. It is noteworthy that statistical differences were only identified within the sylvan environment between L and E (Simpson: *p* = 0.001–0.05; Shannon; *p* = 0.01–0.05) and between L and F0 (Shannon: *p* = 0.01–0.05), while no differences were found between laboratory time points (F2–F10). A significant variation was observed in the transition F0/F2, from wild-caught females to the first time point under laboratory conditions (OTU richness: *p* < 0.05; Shannon: *p* = 0.01–0.05).

**FIGURE 2:**
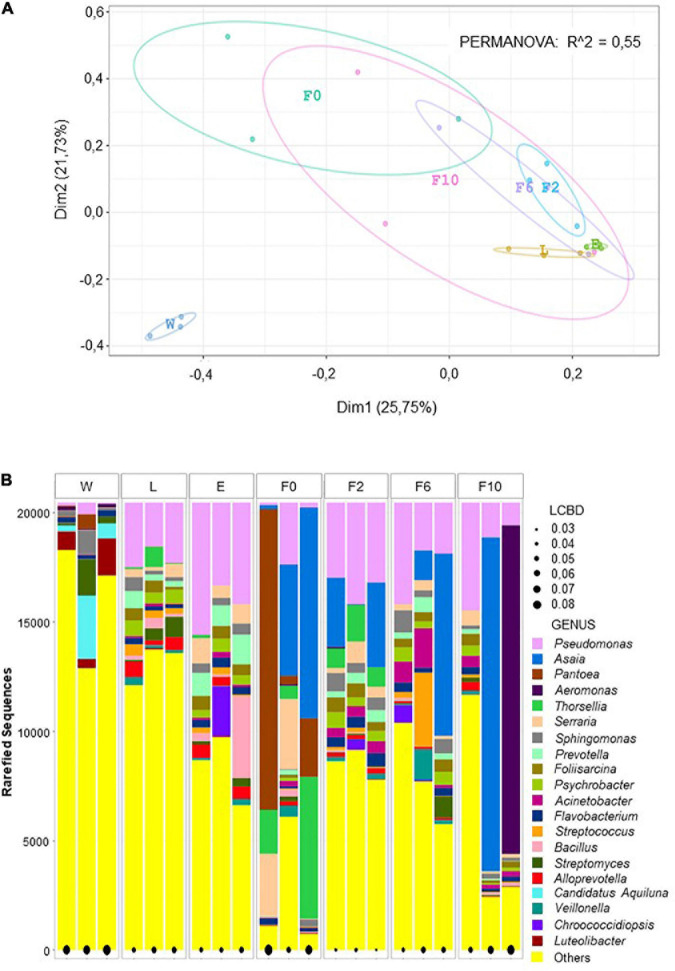
Beta diversity analyses at genus level depicted microbial community variation. PCoA plot showing bacterial community clustering and segregation according to origin. Color points represent the microbiota of a pool of 20 individuals and color ellipses represent confidence intervals per sample type **(A)**. Local contribution to beta diversity analysis (LCBD) showing the uniqueness of bacterial community composition per pool per sample type. The measure of the input is given the size of the black dot (e.g., the larger the dot, the more unique the microbial community) **(B)**. Sample types: W, breeding water; L, larvae; E, newly emerged females; F0, wild-caught females; F2, F6, and F10, laboratory-reared females from the second, sixth, and 10th generations, respectively.

Principal coordinate analyses (PCoA) also evidenced diversity variation among different types of samples and for all taxonomic levels. Spatial distribution and the low variance explained by the first two dimensions (43.5–51.5%) indicated that bacterial communities clustered differently according to origin (i.e., W, L, E, F0–F10). The significance of this differential segregation was further confirmed by PERMANOVA (1,000 permutations; *p* < 0.001; *R*^2^ = 0.53–0.57) (Figure 2A and Supplementary Figures 5A, 6A). When observing ordination plots, it is worth noting that all biological samples (sylvan and laboratory) distributed distantly from natural breeding water (W), suggesting a more unique microbiota composition in the latter, a fact corroborated by LCBD analysis (Figure 2B and Supplementary Figures 5B, 6B). In addition, larvae (L) and newly emerged females (E) distributed closer to laboratory-reared females (F2–F10) than to wild-caught females (F0), implying that, despite their sylvan origin, their microbiota was more similar to that of laboratory females than that of their sylvan counterparts (F0). Furthermore, the heterogeneity previously observed in F0 and F10 (Figure 1) was supported by the extended confidence ellipses shown in the PCoA and by LCBD analysis, which identified these bacterial communities (together with breeding water) as major contributors to the observed diversity differences between sample types (Figure 2B and Supplementary Figures 5B, 6B).

### The Microbiota of *An. atroparvus* Is Governed by *Proteobacteria*, *Firmicutes*, *Bacteroidetes*, and *Actinobacteria*

**FIGURE 3:**
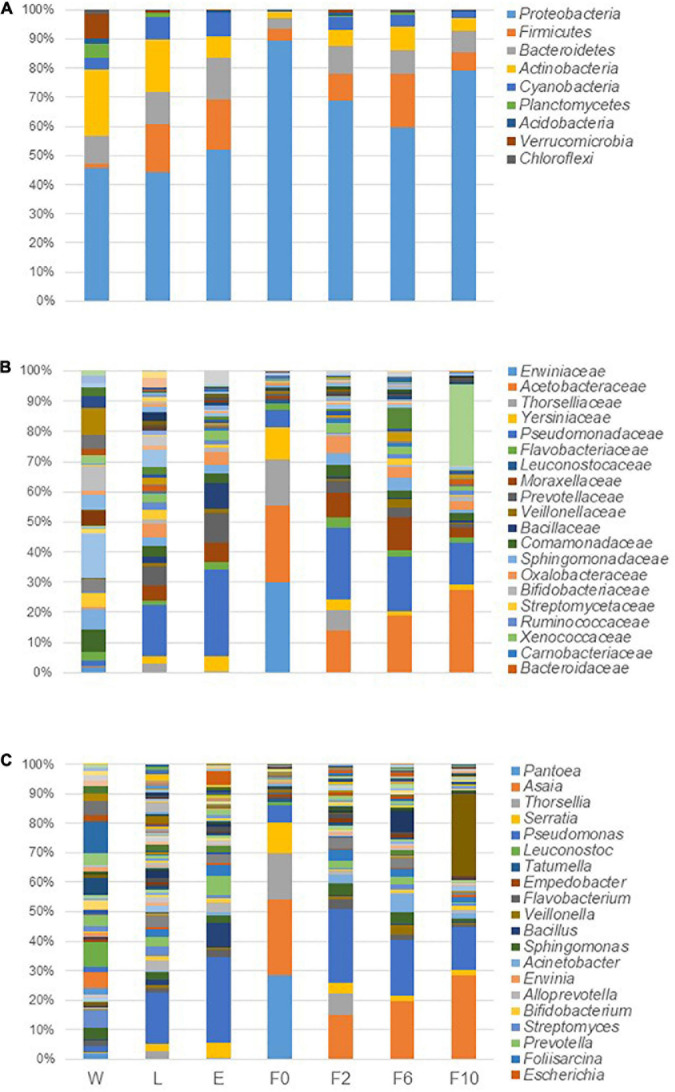
Microbiota composition in breeding water and in sylvan and laboratory-reared *An. atroparvus* at phylum **(A)**, family **(B)**, and genus **(C)** levels. The average of the relative abundances per bacterium from three pools per sample type is represented in bars, and the top 20 taxa are shown.

Taxonomic identification of sequences depicted that the microbiota profile of different sample types analyzed was primarily shaped by the same taxa, although with different proportions (Figure 3). For instance, at phylum level, *Proteobacteria* dominated all bacterial communities with relatively high abundance ranging from 44% in larvae (L) to 89% in wild-caught females (F0). While *Actinobacteria* was the second most abundant phylum in W (22%) and L (18%), it dropped to the third/fourth position in adult females (E and F0–F10) accounting for less than 8% of microbiota. Similarly, whereas *Verrucomicrobia* and *Planctomycetes* belonged to the top five phyla in W, with abundances of 9 and 5%, respectively, these were barely detected in biological samples (<1%). In addition, phyla detected in our studied samples, such as *Firmicutes*, *Bacteroidetes*, and *Cyanobacteria*, also fluctuated between different types of samples below 18, 14, and 8%, respectively (Figure 3A). At lower taxonomic levels, the same trend was observed, and few dominant taxa were identified across sample types. *Pseudomonas* (*Pseudomonadaceae* family), for example, was present in all microbial communities with a high prevalence in biological samples and high abundance ranging from 13% in L and laboratory-reared females at the 10th generation (F10) to 25% in newly emerged females (E). Together with *Pseudomonas*, *Asaia* (*Acetobacteraceae* family) governed the microbiota of laboratory-reared females with frequencies of 12, 17, and 25%, respectively, for F2, F6, and F10; however, in F0, *Pseudomonas* was poorly represented (5%) and in L and E, *Asaia* was scarce (<1%). Besides *Asaia*, the microbiota of F0 was also dominated by *Pantoea* (*Erwiniaceae* family) (27%), *Thorsellia* (*Thorselliaceae* family) (15%), and *Serratia* (*Yersiniaceae* family) (10%), genera that were less frequent in the other sample types (Figures 3B,C). For a more comprehensive profiling, the dominant genera were analyzed at a finer taxonomic level. All the reads associated with *Asaia* belonged to a single species, *Asaia siamensis*, with its most abundant OTU (OTU_19275) being detected in all sample pools including breeding water. Likewise, *Thorsellia anophelis* was the only species recognized with OTU_1682 being found in at least 2 out of three pools of all sylvan samples (including water) and F2. Reads associated with several species of *Pantoea*, *Pseudomonas*, and *Serratia* were identified, although only one OTU assigned to *Pantoea deleyi* (OTU_347) was present in 2 out of three pools of sylvan samples and one OTU assigned to *Pseudomonas migulae* (OTU_107) detected in all sample pools. Lastly, *Serratia liquefaciens* (OTU_1700) was frequently high in almost all sample pools whereas *S. marcescens* was found only in sylvan samples and majorly in F0.

### The Microbiota of *An. atroparvus* Is Acquired Mostly From Its Natural Breeding Water and Can Persist Throughout Different Sylvan Life Stages and Over Laboratory Colonization

**FIGURE 4:**
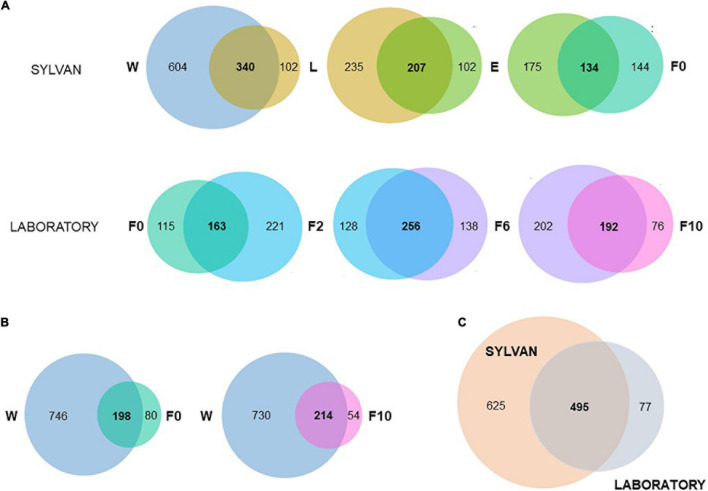
Microbiota of *An. atroparvus* persists across sylvan samples and laboratory time points. Venn diagrams showing the number of shared genera (present in at least one pool, out of three, of both groups) among subsequent pairs **(A)**, between natural breeding water with wild-caught females and with laboratory-reared females from the 10th generation **(B)**, and between sylvan and laboratory environments **(C)**. Sample types: W, breeding water; L, larvae; E, newly emerged females; F0, wild-caught females; F2, F6, and F10, laboratory-reared females from the second, sixth, and 10th generations, respectively.

Pairwise, microbiota comparisons unveiled a considerable fraction of common bacteria between subsequent sample types (Figure 4). Within the sylvan environment, when natural breeding water (W) was contrasted with late-instar larvae (L), more than three-quarters (77%; 340/442) of the bacterial genera detected in L were shared with the water where they developed. Likewise, 67% (207/309) of the genera found in newly emerged females (E) were present in L and 48% (134/278) of bacteria inhabiting wild-caught females (F0) were also identified in E. In the transition from sylvan to laboratory environments, 59% (163/278) of the microbiota found in F0 was recovered in females from the second generation produced under controlled laboratory conditions (F2). During the laboratory colonization process, females from the sixth (F6) and 10th (F10) generation shared 65% (256/394) and 72% (192/268) of their microbiota with their previous time point, F2 and F6, respectively (Figure 4A). It is noteworthy that most bacteria inhabiting F0 and F10 were also identified in natural breeding water (Figure 4B) emphasizing the contribution of the aquatic habitat to the microbiota composition of adult mosquitoes. When sylvan and laboratory samples were compared as a whole, only a small proportion of bacteria were unique to laboratory (Figure 4C). In addition, more than half of the bacteria were unique to the sylvan environment with 64% (398/625) of these bacteria being exclusive to natural breeding water; in larvae, newly emerged and wild-caught females less than 5% of their microbiota was unique.

Finally, through differential abundance analysis, a small fraction of bacteria was considered differentially abundant when subsequent pairs were analyzed (i.e., W/L, L/E, E/F0, F0/F2, F2/F6, and F6/F10). The highest numbers were obtained in transitions W/L (from natural breeding water to late-instar larvae) and F0/F2 (from wild-caught females to laboratory-reared females from the second generation) with 105 and 55 (out of 1197) differential genera, respectively (Supplementary File 2).

### The Core Microbiota of *An. atroparvus* Is Dominated by Few Bacteria

**FIGURE 5:**
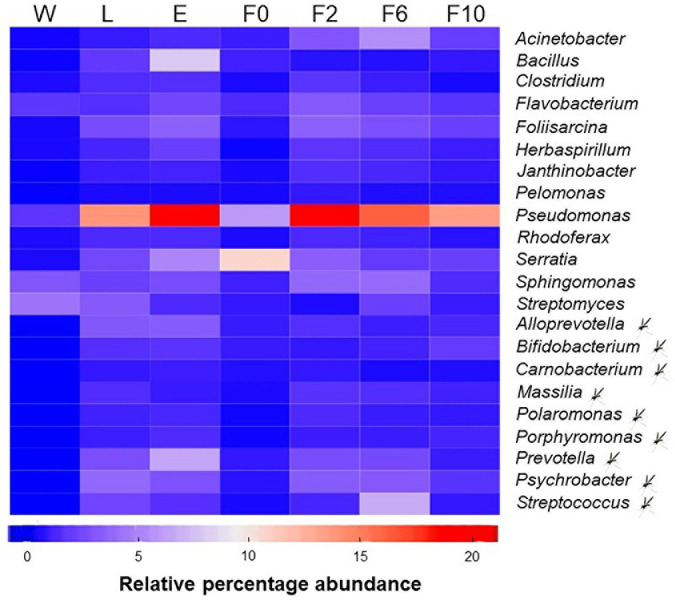
Core microbiota of *An. atroparvus*. Heatmap showing the relative abundance of bacterial genera identified in two out of three pools per sample type with more than 10 reads that are common to all mosquito samples. Genera marked with a mosquito represent core bacteria present in larvae and adult females but not in natural breeding water. Genera without the mark represent core bacteria detected in all sample types. Sample types: W, breeding water; L, larvae; E, newly emerged females; F0, wild-caught females; F2, F6, and F10, laboratory-reared females from the second, sixth, and 10th generations, respectively.

Overall, 22 (out of 1197) bacterial genera were recognized as part of the core microbiota of *An. atroparvus* (Figure 5) with *Pseudomonas* and *Serratia* being the most representative genera shared by immature stages (L) and adult females (E, F0–F10). Thirteen of the core genera in *An. atroparvus* were found in both breeding water and biological samples indicating that these bacteria could have been environmentally acquired. In contrast, the remaining nine were already part of the indigenous microbiota and vertically transmitted.

## Discussion

To set a baseline for future malaria research in *An. atroparvus*, the present study reports, for the first time, the microbiota profile of a sylvan mosquito population from a former malaria transmission area of Europe and assesses field-acquired microbiota along laboratory breeding. Sequencing of the V3-V4 region of the bacterial 16S rRNA gene provided a comprehensive description of bacterial communities and their dynamics across different developmental stages throughout the mosquito’s life cycle and during laboratory colonization under controlled conditions. Our data revealed marked inter-sample variations mostly between sylvan life stages, in the transition from sylvan to laboratory environments, and between the first and last laboratory time points. Overall, these findings suggested that the microbiota of *An. atroparvus* was highly influenced by its breeding habitat (i.e., sylvan or laboratory) and metamorphic processes.

Under natural conditions, throughout their life cycle, mosquitoes are in continuous contact with countless sources of microbes, as well as with unstable extrinsic factors (e.g., temperature, droughts, or heavy rains) that may play a role in shaping their microbiota. *Anopheles* mosquitoes, as holometabolous insects, undergo different developmental stages until complete metamorphosis and so exploit different habitats so as to avoid intraspecific competition (Moran, 1994). In our case, larvae of *An. atroparvus* develop just beneath the water surface of permanent or semi-permanent rice paddies, while adults inhabit terrestrial habitats near domestic animals and human dwellings (Birnberg et al., 2020). Consistent with previous reports in different *Anopheles* and *Aedes* species, water from the aquatic habitat from where larvae were collected exhibited the largest OTU richness, while larvae harbored a higher bacterial diversity than newly emerged and adult females (Wang et al., 2011; Dada et al., 2014; Bascuñan et al., 2018; Alfano et al., 2019). Since immature anophelines are filter feeders, bacteria suspended in the aquatic habitat enter into the gut lumen along with the water intake. Thus, as expected, a substantial fraction of the microbiota recovered in larvae of *An. atroparvus* was present in the water where they develop, confirming high contribution of the aquatic breeding habitat to the microbial community structure in immature stages. Differences in bacterial community structure (diversity and composition) between larvae and their natural breeding water indicated that the larval lumen was the first selective environment for bacteria from the aquatic habitat. While peritrophic matrices work as a physical barrier, the conjunction of the midgut’s physio-chemistry and digestive enzymes, host immune response, and competition with indigenous microorganisms generate a challenging microhabitat in which only a subset of bacteria is able to survive (Engel and Moran, 2013). Microorganisms that withstand and colonize the larval midgut are presumed to offer functional advantages to their hosts (Gimonneau et al., 2014). For instance, *Actinobacteria*, which are environmentally derived bacteria, were highly prevalent in *An. atroparvus* larvae and persistent in the adult population. Due to the association of *Actinobacteria* to plant biomass decomposition in aquatic environments (Lewin et al., 2017), these bacteria could be associated with *An. atroparvus* nutritional functions as suggested for other anophelines from Colombia (Bascuñan et al., 2018).

In the transition from aquatic to terrestrial habitats, metamorphosis from larvae to adults involves selective processes that modify the structure of the microbiota. During the ecdysial process, the egestion of the meconial peritrophic matrices (MPMs) and the eventual ingestion of exuvial fluid (with its antiseptic properties) clear the midgut content (Moll et al., 2001), drastically reducing the microbial communities (Wang et al., 2011). Accordingly, the shift from larvae to newly emerged females in *An. atroparvus* resulted in a significant diversity loss, albeit a fraction of the bacterial communities persisted and shared by both developmental stages. This finding is in agreement with previous studies that have analyzed the microbiota dynamics throughout the life cycle of several mosquito populations and reported also in microbial persistence among subsequent stages, suggesting bacterial transstadial transmission (Rani et al., 2009; Coon et al., 2014, 2016; Gimonneau et al., 2014). In our study, bacterial persistence could have implied one and/or a combination of the following phenomena: (i) an incomplete egestion of MPMs (Moll et al., 2001) and (ii) MPMs that were still present in newly emerged females, due probably to the age of the studied specimens. Data herein reported derived from 0- to 48-h-old newly emerged females and the disappearance of MPMs in *Anopheles* mosquitoes has been seen to occur 16–20 h after emergence (Romoser et al., 2000). (iii) Part of the bacteria could have been reacquired by newly emerged females by imbibing water during hatching (Lindh et al., 2008), since pupae from which *An. atroparvus* females emerged were maintained in their original breeding water. (iv) Bacteria that were transmitted by transstadial means colonized other tissues that are not affected by the potential antibacterial effect of the exuvial (molting) fluid, which may be ingested during metamorphosis (Moll et al., 2001). The high overlap between the bacterial communities in larvae and newly emerged *An. atroparvus* females, which had not been sugar fed, reflected the contribution of the larval aquatic environment to adults’ microbiota as previously reported for other anophelines (Akorli et al., 2016), highlighting the relevance of microbial transstadial transmission in shaping the community structure of adult *An. atroparvus* females. Aside from the influence of metamorphosis in the structure of bacterial communities during the shift from aquatic to terrestrial habits, physiological requirements of adult females involve behavioral and nutritional changes that may also alter their microbiota. Immediately after emergence, adult females predominantly feed on nectar or honeydew to satisfy energetic flight requirements and may introduce diversity and/or favor the proliferation of certain bacteria (Buck et al., 2016). In the present study, *Asaia* which is an acetic acid bacterium could have been horizontally acquired from flower nectar as has already been demonstrated for anopheles mosquitoes (Bassene et al., 2020), or growth could have been enhanced by sugar ingestion, since it was scarce in larvae and newly emerged females, while in wild-caught *An. atroparvus* females it was highly abundant. Moreover, adult females also ingest blood to fulfill protein requirements for oviposition. Blood digestion produces several changes in internal midgut conditions, which may limit the growth of certain bacteria while enhancing the expansion of others (Wang et al., 2011; Sharma et al., 2020). Accordingly, in *An. atroparvus*, a significant decline in diversity was observed in wild-caught females with the dominance of few bacteria that have been previously reported to succeed during blood digestion, such as *Thorsellia*, *Pantoea*, and *Serratia* (Briones et al., 2008; Wang et al., 2011, 2012; Akorli et al., 2016). Despite the feeding history of wild-caught females in our study being unknown, blood feeding could be evidenced by the gravid status following the inspection of a subset of females from the same cohort (Birnberg et al., 2020). Unexpectedly, *Pseudomonas* which has been observed to proliferate in the presence of blood (Wang et al., 2011; Sharma et al., 2020) showed an attenuated abundance in wild-caught *An. atroparvus* females, probably blood-fed, a fact that would require further investigation. As evidenced, and consistent with other reports on culicid mosquitoes (Boissiere et al., 2012; Osei-Poku et al., 2012), wild-caught *An. atroparvus* females harbored low diversity but highly variable bacterial communities. This high variation supported the dominant role of the environment in determining the microbiota in adult mosquitoes. Environmentally derived gram-negative bacteria associated with soil, water, plants, and animals dominated the microbiota of *An. atroparvus*, the vast majority from the phylum *Proteobacteria*. Most of the bacterial taxa herein reported have been described as part of the microbiota in culicid mosquitoes (Dada et al., 2014; Muturi et al., 2016; Rocha-David et al., 2016; Hegde et al., 2018; Kang et al., 2020) including *Anopheles* from different geographic regions (Rani et al., 2009; Djadid et al., 2011; Wang et al., 2011; Boissiere et al., 2012; Gimonneau et al., 2014; Ngo et al., 2015; Bogale et al., 2020; Galeano-Castañeda et al., 2020; Zoure et al., 2020; Feng et al., 2021; Silva et al., 2021).

Finally, to achieve an established colony, laboratory breeding constituted a further shift of breeding habitat, which influenced the structure of microbial communities associated with *An. atroparvus*. Contrary to what occurs in the sylvan environment, the life cycle of the mosquito in the laboratory develops under controlled environmental conditions and is dependent always on the same type of food. Herein, immature stages were maintained in clean dechlorinated tap water and fed an equal amount of balanced fish:turtle food, while adults were offered sterile sucrose and rabbit-blood meals for daily maintenance and oviposition purposes, respectively. It has been suggested that the periodic use of dechlorinated tap water and standard protocols for rearing laboratory colonies have been the cause of diversity loss even among early generations (Akorli et al., 2019; Dada et al., 2020). Conversely, in the transition from wild-caught *An. atroparvus* females to the first laboratory time point analyzed, a significant increase of diversity was observed in laboratory-reared females from the second generation (F2). Interestingly, similar findings were observed only when *Anopheles gambiae* were reared using field-larval water to preserve its field-derived microbiota (Akorli et al., 2019). The bacterial increase in F2 *An. atroparvus* might be linked to a closer relationship with bacteria acquired from their larval breeding habitat, which could have been transiently masked by the dominance of certain taxa acquired and/or proliferated, circumstantially, in wild-caught females due to their physiological needs and/or foraging habits (Buck et al., 2016). This fact could be supported by the similitude of the microbial composition associated with F2 with that of larvae and newly emerged females.

In the following laboratory generations, and consistent with previous studies of other mosquito species (Rani et al., 2009; Coon et al., 2014; Dickson et al., 2018; Akorli et al., 2019), a continuous decline in bacterial diversity was observed in *An. artroparvus* females, although no significant variation was identified up until the 10th generation. This low diversity variation within laboratory colonies may be attributed to standard laboratory conditions and uniform physiological traits in laboratory specimens as previously suggested for *Ae. albopictus* and *An. gambiae* (Minard et al., 2018; Akorli et al., 2019).

Conservation of numerous environmentally acquired bacterial taxa up until the 10th generation, not only suggests the evolutionary conservation of symbiotic associations of *An. atroparvus* with indigenous bacteria but also evidences the presence of a core microbiota, which may contribute basic information for developing better-adapted vector and disease control strategies. Identifying core symbionts may facilitate the selection of para-transgenesis candidates for interference with pathogen transmission (Wilke and Marrelli, 2015), the generation of axenic/gnotobiotic mosquito models to investigate the effects of the microbiome on mosquito biology without the use of antibiotics (Steven et al., 2021), as well as finding probiotics to improve key factors for population suppression techniques, such as mating performance, mass production, and longevity of sterile males (Chen et al., 2020). In the present study, finding *Serratia* as part of the core microbiota of *An. atroparvus* is promising for local malaria control as *S. marcescens* can reduce mosquito survival, influence the susceptibility of *Anopheles* mosquitoes to *Plasmodium* infections, and decrease parasitical loads (Bando et al., 2013; Bahia et al., 2014; Bai et al., 2019). In fact, *S. liquefaciens* has already been identified as a cultivable bacterium from *An. darlingi* midgut (Arruda et al., 2021), the first step for para-transgenesis. However, it is worth noting that, *S. marcescens* was lost from *An. atroparvus* females during laboratory colonization, a fact that should be further analyzed since it could affect its suitability for para-transgenesis in the studied population. In addition, *Pseudomonas*, identified as the most abundant core genus in *An. atroparvus*, opens up new perspectives for control approaches since it has been suggested as an appropriate candidate for para-transgenesis (Raharimalala et al., 2016), although its role in the biology and vector competence of *An. atroparvus* still needs to be investigated. Furthermore, the high prevalence of *Asaia* in sylvan and laboratory-reared females emphasized its potential use for prevention of malaria in the future and for vector control strategies in Southern Europe. *Asaia* has been proposed as being the most suitable candidate for para-transgenic approaches as it gathers the ecological (e.g., associated with diverse mosquito species; colonizes the midgut, salivary glands, and reproductive organs; horizontally and vertically transmitted), immunological (e.g., production of anti-plasmodial effector molecules), and technical (e.g., cell-free culture, genetically transformable) requirements for this approach (Favia et al., 2007, 2008; Damiani et al., 2010; Strand, 2017; Rami et al., 2018). Moreover, the high prevalence of *Asaia* in *An. atroparvus* females could explain the absence of *Wolbachia*, as previously described for other *Anopheles* natural populations (Rossi et al., 2015).

To conclude, our study constitutes the first report of the microbiota associated with a sylvan *An. atroparvus* population and significantly contributes to the knowledge of malaria vectors in Europe. Our findings confirm the key role of the breeding environment in shaping the microbiota of vector species and corroborate the decline in diversity during laboratory colonization. The identification of a core microbiota in *An. atroparvus* is a relevant finding that highlights evolutionary conservation of association with its resident bacteria and focuses attention on a limited number for para-transgenic use. Data herein reported may well contribute in creating a well-defined microbiome baseline for further studies on the effects of microbiome manipulation on mosquito phenotypes for malaria research purposes.

## Data Availability Statement

The datasets presented in this study can be found in online repositories. The names of the repository/repositories and accession number(s) can be found in the article/Supplementary Material.

## Author Contributions

NB, LB, and FC conceived the experimental design. NB acquired the funding and administered the project. LB collected and prepared the sample pools for microbiome analysis. FC and EC-S performed the sample sequencing and bioinformatic analysis. LB and NB prepared the original draft of the manuscript. All authors conducted the data analyses, read, and agreed the final version of the manuscript.

## Conflict of Interest

The authors declare that the research was conducted in the absence of any commercial or financial relationships that could be construed as a potential conflict of interest. The reviewer MG declared a shared affiliation, with the authors to the handling editor at the time of the review.

## Publisher’s Note

All claims expressed in this article are solely those of the authors and do not necessarily represent those of their affiliated organizations, or those of the publisher, the editors and the reviewers. Any product that may be evaluated in this article, or claim that may be made by its manufacturer, is not guaranteed or endorsed by the publisher.

## Footnotes

1
http://www.exelixis-lab.org/web/software/pear
