# Supplementary material for: Microbiota Variation Across Life Stages of European Field-Caught Anopheles atroparvus and During Laboratory Colonization: New Insights for Malaria Research
Source: Front Microbiol. 2021 Nov 24;12:775078. doi: 10.3389/fmicb.2021.775078 (PMC8652072; doi:10.3389/fmicb.2021.775078)
Supplement: Supplementary file 3 [file Data_Sheet_1.docx]

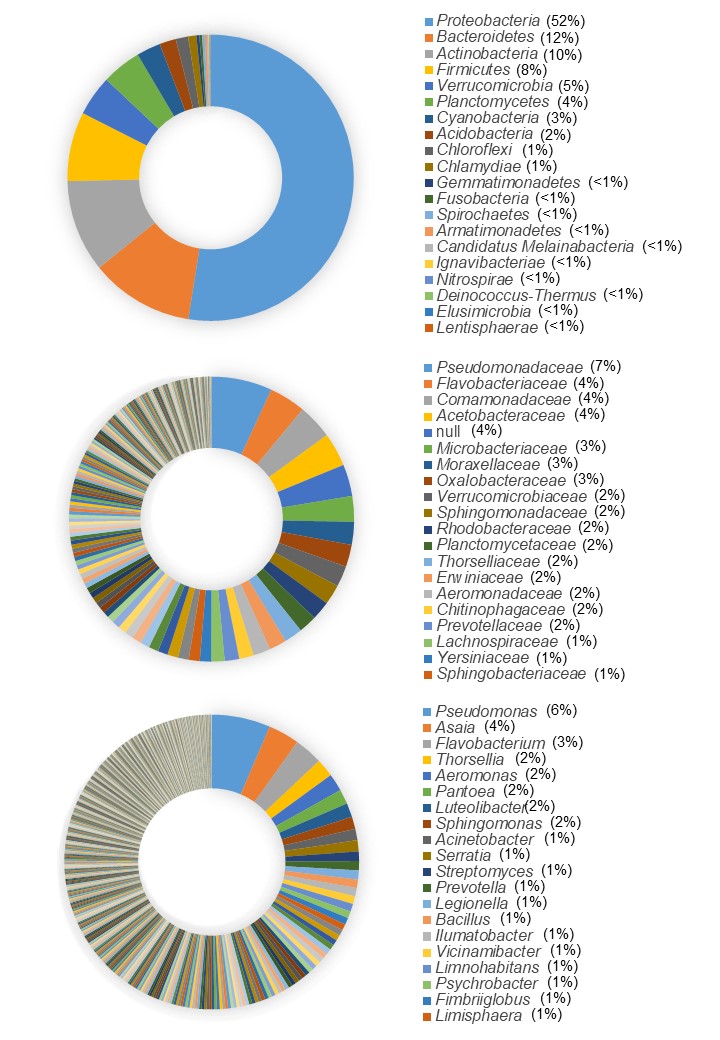


(C)

(B)

(A)

**Supplementary Figure 1.** OTU diversity distribution. Donut charts showing the percentage of OTUs annotated at phylum **(A)**, family **(B)** and genus **(C)** levels. OTUs identified as “null” represent taxa that were not classified at the given taxonomic level but their classification could be found at lower or higher levels.
