# Supplementary material for: Microbiota Variation Across Life Stages of European Field-Caught Anopheles atroparvus and During Laboratory Colonization: New Insights for Malaria Research
Source: Front Microbiol. 2021 Nov 24;12:775078. doi: 10.3389/fmicb.2021.775078 (PMC8652072; doi:10.3389/fmicb.2021.775078)
Supplement: Supplementary file 4 [file Data_Sheet_2.docx]

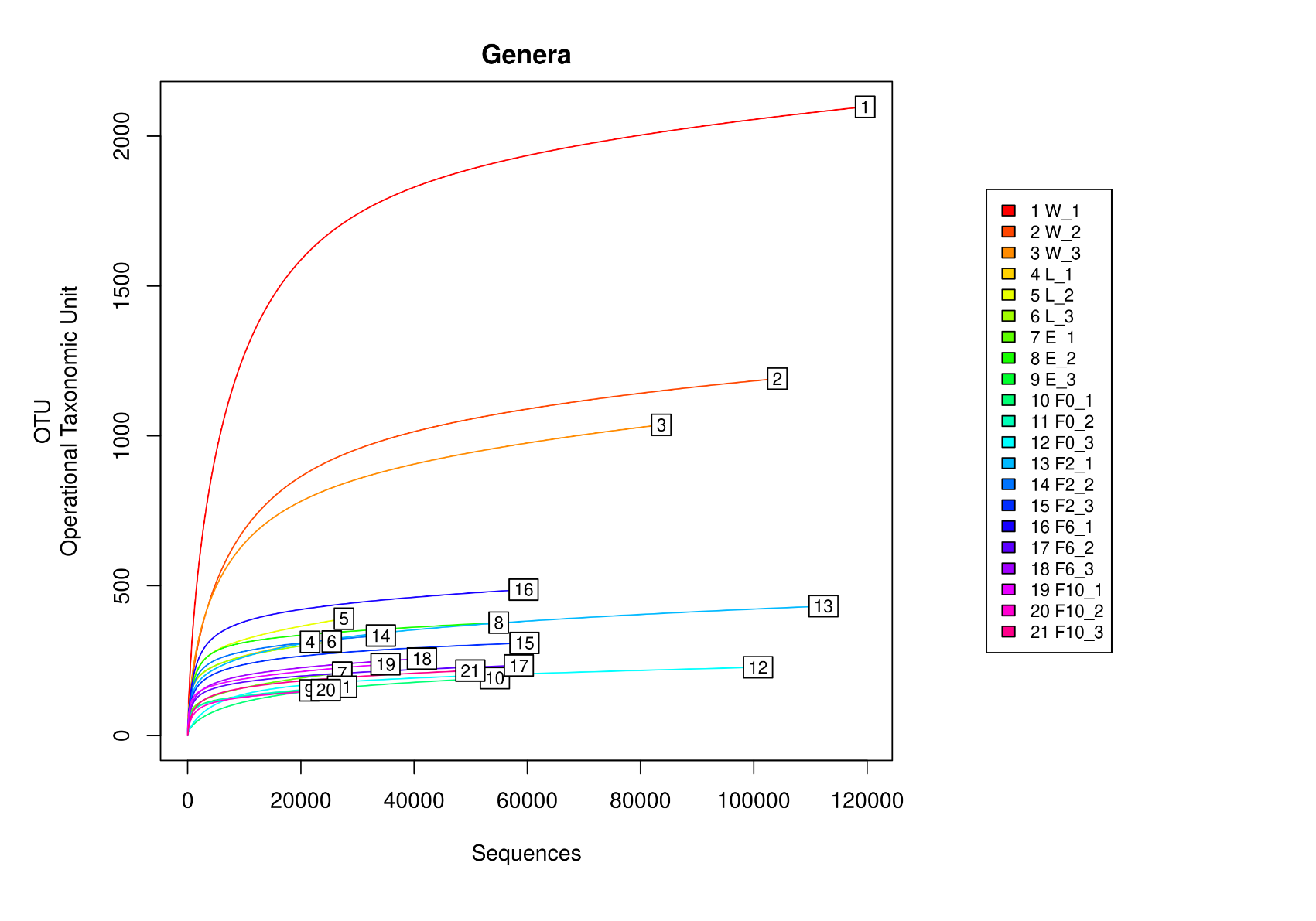


**Supplementary Figure 2.** Rarefaction curves showing that most of the samples reached the plateau suggesting that the majority of genera were captured at the sequencing depth.
