# Supplementary material for: Microbiota Variation Across Life Stages of European Field-Caught Anopheles atroparvus and During Laboratory Colonization: New Insights for Malaria Research
Source: Front Microbiol. 2021 Nov 24;12:775078. doi: 10.3389/fmicb.2021.775078 (PMC8652072; doi:10.3389/fmicb.2021.775078)
Supplement: Supplementary file 5 [file Data_Sheet_3.docx]

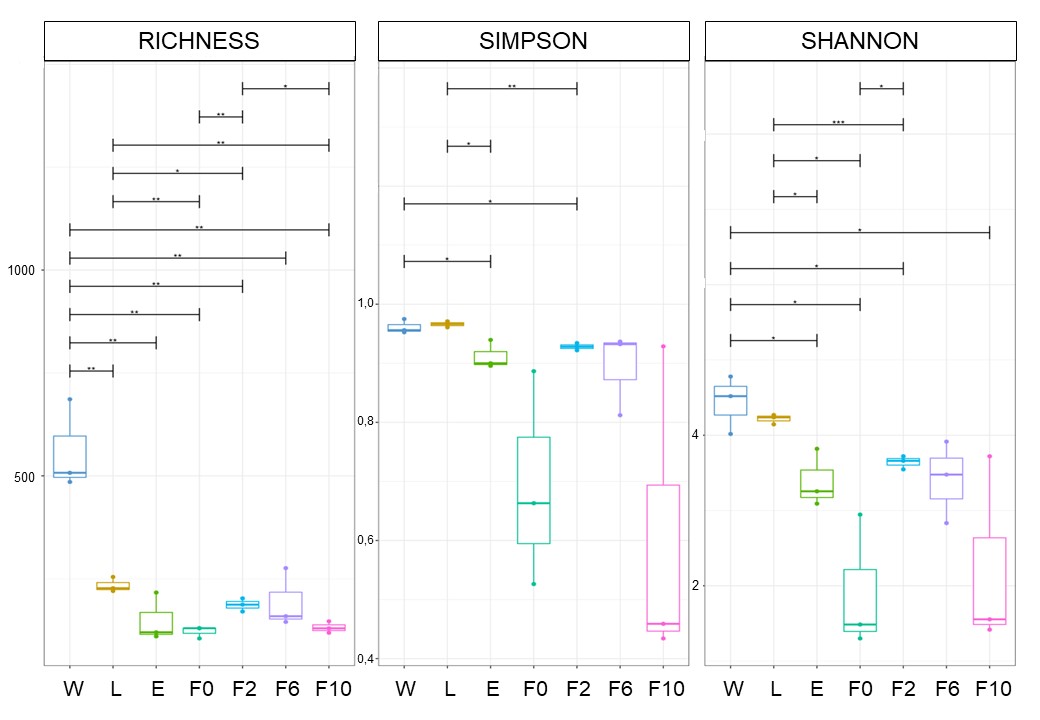


**Supplementary Figure 3.** Differences in bacterial community structure. OTU richness, Simpson and Shannon indices estimated at genus level. Sample types: W, breeding water; L, larvae; E, newly emerged females; F0, wild-caught females; F2, F6 and F10, laboratory-reared females from the second, sixth and tenth generation respectively. Boxes represent the interquartile range within each group. The line that divides the box corresponds to the median and dots, to minimum and maximum scores. Analysis of Variance (ANOVA) significance levels: * p = 0.01 – 0.05; ** p = 0.001 – 0.01; *** p < 0.001.
