# Supplementary material for: Microbiota Variation Across Life Stages of European Field-Caught Anopheles atroparvus and During Laboratory Colonization: New Insights for Malaria Research
Source: Front Microbiol. 2021 Nov 24;12:775078. doi: 10.3389/fmicb.2021.775078 (PMC8652072; doi:10.3389/fmicb.2021.775078)
Supplement: Supplementary file 8 [file Data_Sheet_6.docx]

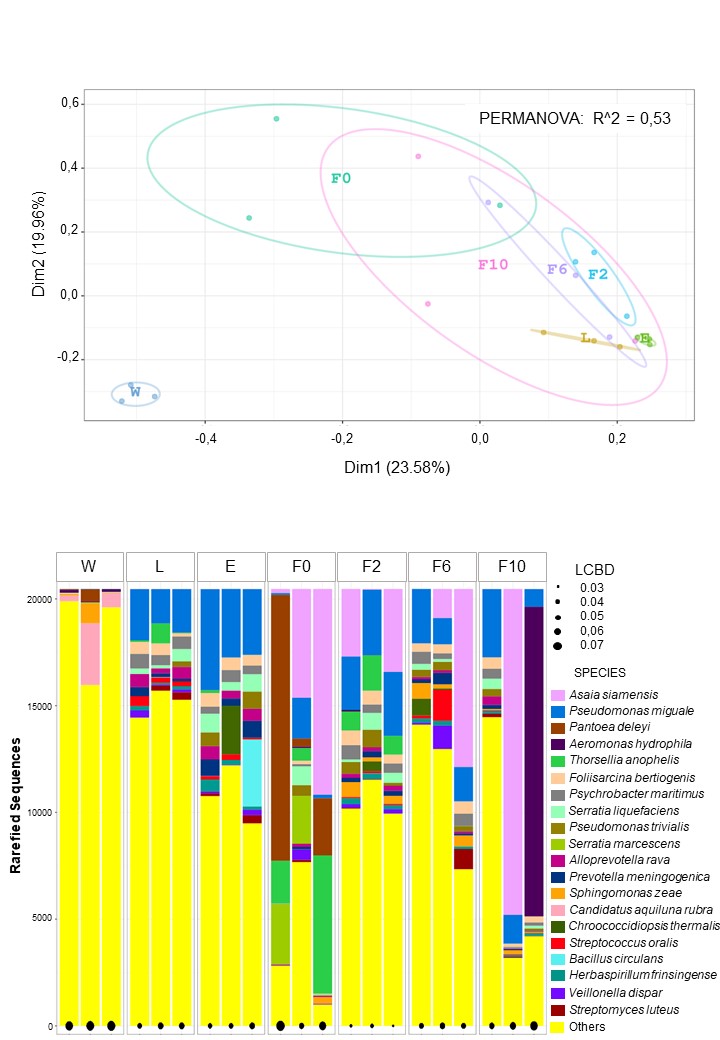


(A)

(B)

**Supplementary Figure 6.** Beta diversity analyses at species level depicted microbial community variation. PCoA plot showing bacterial community clustering and segregation according to origin. Color points represent the microbiota of a pool of 20 individuals and color ellipses represent confidence intervals per sample type **(A)**, Local contribution to beta diversity analysis (LCBD) showing the uniqueness of bacterial community composition per pool per sample type. The measure of the input is given the size of the black dot (e.g., the larger the dot, the more unique the microbial community) **(B)**. Samples types: W, breeding water; L, larvae; E, newly emerged females; F0, wild-caught females; F2, F6 and F10, laboratory-reared females from the second, sixth and tenth generation respectively.
